# Cellular Interaction of Bone Marrow Mesenchymal Stem Cells with Polymer and Hydrogel 3D Microscaffold Templates

**DOI:** 10.1021/acsami.1c23442

**Published:** 2022-03-13

**Authors:** Beatriz N. L. Costa, Ricardo M. R. Adão, Christian Maibohm, Angelo Accardo, Vanessa F. Cardoso, Jana B. Nieder

**Affiliations:** † INL—International Iberian Nanotechnology Laboratory, Ultrafast Bio- and Nanophotonics Group, Av. Mestre José Veiga S/n, 4715-330 Braga, Portugal; ‡ CMEMS-UMinho, University of Minho, DEI, Campus de Azurém, Guimarães 4800-058, Portugal; § Faculty of Mechanical, Maritime, and Materials Engineering (3mE), Department of Precision and Microsystems Engineering (PME), Delft University of Technology, Mekelweg 2, Delft 2628 CD, The Netherlands; ∥ CF-UM-UP, Centro de Física das Universidades do Minho e Porto, Universidade do Minho, Campus de Gualtar, 4710-057 Braga, Portugal

**Keywords:** two-photon polymerization, three-dimensional scaffolds, woodpile structures, polymer, hydrogel, bone marrow mesenchymal stem cells, tissue engineering

## Abstract

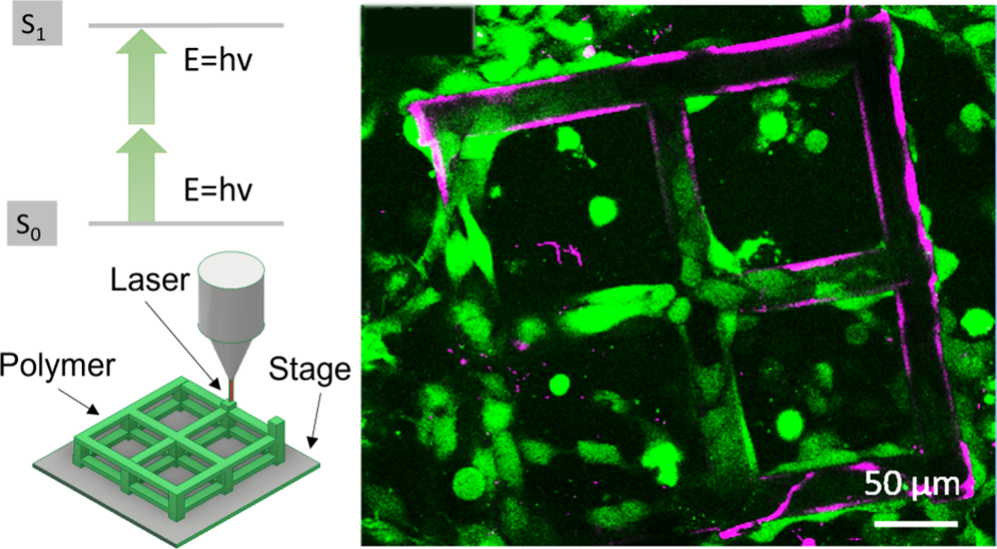

Biomimicking biological niches of healthy tissues or tumors can be achieved by means of artificial microenvironments, where structural and mechanical properties are crucial parameters to promote tissue formation and recreate natural conditions. In this work, three-dimensional (3D) scaffolds based on woodpile structures were fabricated by two-photon polymerization (2PP) of different photosensitive polymers (IP-S and SZ2080) and hydrogels (PEGDA 700) using two different 2PP setups, a commercial one and a customized one. The structures’ properties were tuned to study the effect of scaffold dimensions (gap size) and their mechanical properties on the adhesion and proliferation of bone marrow mesenchymal stem cells (BM-MSCs), which can serve as a model for leukemic diseases, among other hematological applications. The woodpile structures feature gap sizes of 25, 50, and 100 μm and a fixed beam diameter of 25 μm, to systematically study the optimal cell colonization that promotes healthy cell growth and potential tissue formation. The characterization of the scaffolds involved scanning electron microscopy and mechanical nanoindenting, while their suitability for supporting cell growth was evaluated with live/dead cell assays and multistaining 3D confocal imaging. In the mechanical assays of the hydrogel material, we observed two different stiffness ranges depending on the indentation depth. Larger gap woodpile structures coated with fibronectin were identified as the most promising scaffolds for 3D BM-MSC cellular models, showing higher proliferation rates. The results indicate that both the design and the employed materials are suitable for further assays, where retaining the BM-MSC stemness and original features is crucial, including studies focused on BM disorders such as leukemia and others. Moreover, the combination of 3D scaffold geometry and materials holds great potential for the investigation of cellular behaviors in a co-culture setting, for example, mesenchymal and hematopoietic stem cells, to be further applied in medical research and pharmacological studies.

## Introduction

Tissue engineering (TE) is a vast field that applies the principles of engineering and life sciences toward the development of biological substitutes that restore, maintain, or improve tissue function of a whole organ.^1^ The in vitro TE approach can be divided into four steps, namely, fabrication of biomaterial scaffold, scaffold seeding with primary or stem cells, culture in a bioreactor, and finally implantation.^2^ Scaffolds are among the most promising environments promoting cell growth and the consequent tissue formation, providing porous three-dimensional (3D) support structures that mimic the extracellular matrix (ECM).^3−5^ These structures provide an alternative to the currently limited two-dimensional (2D) in vitro cell models that do not consider crucial natural environment features, such as non-continuous nutrients’ access and spatial organization. The 2D approaches are limited in their prediction of cell–drug interactions, which causes the need for extended in vivo studies on animal models and clinical samples alike. Cellular interactions with scaffold architectures require interdisciplinary advances of material science, 3D fabrication technologies, and in vitro assays capable of assessing biocompatibility and tissue growth.^6−8^ In fact, 3D structures stand out due to their capability to recreate complex volumetric geometries with minimal toxicity and favor cell–biomaterial interactions as well as nutrient exchange.^9^ One widespread design used in 3D scaffolds is the denominated woodpile, which provides complexity, pores to support migration, and large surface areas for cells to grow on, allowing the specific study of cell in-growth and effects of the pore size.

These structures can be fabricated using a wide range of materials and recently developed techniques.^10^ Following the definition of the American National Institute of Health, a biomaterial is a substance with capabilities of augmenting or replacing, partially or not, any tissue, organ, or function of the body and can have different origins.^11^ Biomaterials are commonly divided into natural protein/polymeric materials and synthetic materials. The first category originates from ECM components [e.g., fibrin, collagen-I,^12^ gelatin (Gel),^13^ etc.]. In the synthetic materials’ category, we can mention poly(ethylene glycol) and derivates,^13,14^ polylactic acid,^15^ IP-resins,^16^ SZ2080,^17^ and so forth. Naturally, the choice of material depends not only on the mechanical properties and the target cell line but also on the manufacturing technique, sometimes characterized by a limited range of compatible materials. Mechanical properties, namely stiffness, are important since each tissue has a specific reference value intrinsically associated to its function and exposure to mechanical loading.^18^ Besides, the combination of scaffold stiffness and pore size has been proven to be one of the most determining factors for cell invasion and cancer progression, supporting the importance of both these properties.^19,20^

The fabrication of 3D microenvironments requires techniques that allow the accurate and reproducible 3D manufacturing of scaffolds. The scaffold manufacturing techniques can be mainly divided into conventional fabrication techniques and addictive manufacturing (AM) ones.^21,22^ The first category includes less costly but also less precise techniques, while the second one requires expensive processes, which, on the other hand, manage to achieve geometries with a higher complexity. Solvent casting/particle leaching, gas foaming, freeze-drying, and electrospinning are some of the main methods conventionally used to obtain scaffolds.^12,23,24^ The most investigated AM technologies span over fused deposition modeling, stereolithography, selective laser sintering, and ink-jet printing, among other printing systems.^10,25^

Another emerging rapid prototyping technique for 3D microscale architectures resorts to femtosecond laser-based direct laser-writing (DLW) and is known as two-photon polymerization (2PP). In the 2PP process, light absorption is a nonlinear photochemical process that decays quadratically over the distance from the focus point, thus confining the writing voxel and leading to a spatial resolution down to hundreds of nanometers that far exceeds the capabilities of conventional DLW techniques. Furthermore, it is compatible with a wide range of photosensitive materials.^10,24^ The 2PP systems are also known for their ability to fabricate very complex features and designs.^26−28^ Recently, 2PP-fabricated scaffolds have been used to cultivate different cell lines.^13,29,30^ In 2013, Raimondi et al.^31^ were the first who developed, using 2PP, SZ2080 scaffolds resembling the mesenchymal stem cell (MSC) niche to support and guide cell growth, where evident cell proliferation in the niches was observed. After 1 year, Raimondi et al.^32^ reported the niche optimization, observing direct stem cell homing and colony formation, guided aggregate formation, and space for cells to adhere and renew. The use of IP-resins and poly(ethylene glycol)diacrylate (PEGDA) materials showed promising results with human epithelial cell lines and neurons.^16,33,34^

MSCs are part of the large group of stem cells from which many human body cells take origin. They are characterized by self-maintenance and self-renewal abilities, as well as their plastic-adherence spindle shape. Their trilineage mesenchymal differentiation characteristic is essential to explore these cells’ tissue regeneration capabilities and differentiation processes.^35^ Several studies claim that bone marrow mesenchymal stem cells (BM-MSCs) are the primordial regulators of hematopoietic stem cells and play a fundamental role in regulating leukemogenesis, a process that disturbs normal blood homeostasis.^35,36^ BM-MSCs are deeply related to hematological processes, influencing the treatment and prevention of diseases linked to blood and immune system diseases, such as leukemia, myeloma, and lymphoma.^35^ Therefore, it is thought that fighting hematological diseases through novel therapies is interconnected with tuning BM-MSCs’ influence on the BM microenvironment.^35^ Osteogenesis imperfecta, infantile hypophosphatasia, osteoporosis, osteoarthritis, and rheumatoid arthritis are also potential therapeutic applications of MSCs in bone diseases.^37^ The BM is a very complex microenvironment in charge of maintaining the stem cell nature of BM-MSCs. To recreate this specific niche and study BM-MSCs’ implications in different biological phenomena, keeping the cells’ original features without differentiation predisposition is essential.^38^ A 3D approach allows transcending from 2D cultures that fail in mimicking migration, cell spatial disposal, interactions, and nutrients’ exchange.

In this work, we aim to reach the first two steps of the in vitro TE approach, namely, scaffold fabrication and cell seeding, providing an enhanced and biomimicking in vitro model closer to a realistic 3D ECM, which may be used for medical research applications. Here, the manufacture and optimization of 3D scaffolds based on woodpile structures from different materials, namely, IP-S, SZ2080, and PEGDA 700 using 2PP are reported. These scaffolds are designed with a constant beam diameter of 25 μm and a gap-size considering the BM-MSCs’ size. The scaffold design considers previous studies performed with BM-MSCs, showing that these cells are more likely to maintain the proliferative and bilineage differentiation potential in a 3D woodpile design.^39^ Increasing scaffold’s similarity to the BM niche by the presence of BM-MSCs and fibronectin glycoprotein paves the way to study various hematological cancers, both in terms of their biological behavior and toward personalized therapies.^36^ After completing the fabrication, the cells are grown on the woodpile scaffolds in conventional cell culture systems. A complementary study was also performed using HeLa cells to test the biocompatibility of the fabricated scaffolds with a cancer cell line. The evaluation of cell growth and proliferation with these two different cell lines shows the capability of these scaffolds to host different cell lines, carcinogenic or not, and to become a platform for further mechanobiological and drug screening studies.

## Results and Discussion

### Optimization of the 2PP Fabrication Process

The main factors that affect the overall mechanical stability of a 2PP structure are its design, material, and the writing parameters, which influence the cross-linking process. Writing parameters leading to stable polymerization even in challenging 3D “microgrid” structures composed of relatively thin support beams should ensure mechanically stable woodpile structures composed of much larger diameter beams that require a longer fabrication time. The optimization was performed in terms of writing speed (*v*_WS_) and laser power (*P*_L_).

Succinctly, a v_WS_ below 15 mm·s^–1^ and a *P*_L_ above 35 mW led to an improved stability but a lower resolution. Figure S1 shows scanning electron microscopy (SEM) images regarding the optimization of *v*_WS_ and *P*_L_ parameters for IP-DIP material. Similar optimization procedures have been previously reported elsewhere.^40^

IP-DIP and IP-S are very similar in terms of chemical composition and mechanical properties, such that the microgrid optimization results were fundamental to facilitate and accelerate the IP-S 3D scaffolds’ fabrication and optimization (see further details of the optimization process in Figures S1 and S2).

### Fabrication of the 3D Woodpile Structures

**Figure 1:**
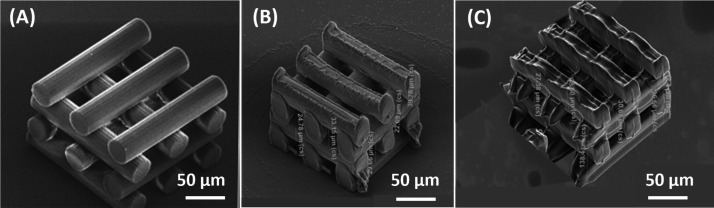
Representative SEM images of optimized woodpile structures with a 25 μm gap following the same design: (A) IP-S, (B) SZ2080, and (C) PEGDA 700. Images were taken at 45°, 10 kV, ×700 magnification.

The fabrication protocol, involving both a commercial and a customized 2PP setup (see experimental section), of the woodpile structures based on the polymers IP-S and SZ2080 and the hydrogel PEGDA 700 was defined according to the distance between the beams (gap size), that is, 25, 50, and 100 μm, keeping a fixed diameter equal to 25 μm. The spacing was chosen according to the cells’ size, and it was defined to understand how different gap sizes influence the cell behavior. The average MSC diameter is reported to be between 18 and 31 μm,^41^ and HeLa cells have a diameter of approximately 17 μm.^42^ The fixed beam diameter of 25 μm is an intermediate size of BM-MSCs and, at the same time, thick enough to provide mechanical stability to the woodpile design, especially for the softer material, PEGDA 700. The materials required optimizations that involved laser intensity, writing speed, and voxel shape compensation. Figure 1 shows the SEM images of the optimized woodpile structures of the three materials, with a 50 μm gap, as representative examples. The results obtained for each material are analyzed in detail in the following.

The optimized IP-S structures are obtained at a writing speed of 100 mm·s^–1^ and maximum *P*_L_ (50 mW). In the chemical development phase, 15 min of immersion in propylene glycol methyl ether acetate (PGMEA) and a 30 s Novec rinse are performed. The IP-S woodpile structures did not present overexposure problems, precisely following the designed dimensions and showing smooth lines (Figure 1A). Also, 3D SZ2080 woodpile structures fabricated using the 2PP customized setup (Figure S3A) showed dimensions identical to the designed ones except for the edges of the cylinders, where the base points coincide with the final printed circumference and overexposure of the material (Figure 1B). The beams also presented some marks, exhibiting the connection points between unit structures (circles). The developed structures with no signs of detachment indicate the suitability for cell interaction studies. The woodpile structures’ fabrication with PEGDA 700 was more challenging and required a lower numerical aperture (NA) objective as well as a voxel-size design compensation to achieve the structures shown in Figure 1C. The NA value is defined by the product between the one-half angular aperture of the objective and the medium’s refractive index between the objective front lens and the specimen. The lower magnification objective has a lower NA, leading to an increased voxel size and to the augmentation of the laser beam distortion factor.^43^ The performed simulations (see Figure S4) helped to decrease and compensate for the impact of the microscope objective on the voxel size through design modifications. In contrast to the other stiffer polymeric materials, small irregularities are found in the beams of PEGDA 700 structures, which is not necessarily a detrimental outcome since it has been proved that surface roughness may contribute to cell attachment.^44^ This behavior occurs likely because of the mechanical instability of the softer materials and the use of a bottom-up fabrication process, where the lower layers can partially block or shift the laser beam during the writing of the upper ones (air printing mode). The schematic in Figure S3B shows the differences between the two working configurations, oil immersion, and dip-in laser lithography (DiLL) mode. Micro-rugosities can still be found in the other two structures, even if at a smaller scale. This is naturally introduced by the fabrication process, excluding the need to incorporate these features into the design and increase the fabrication times.

### PEGDA 700 Mechanical Properties

**Figure 2:**
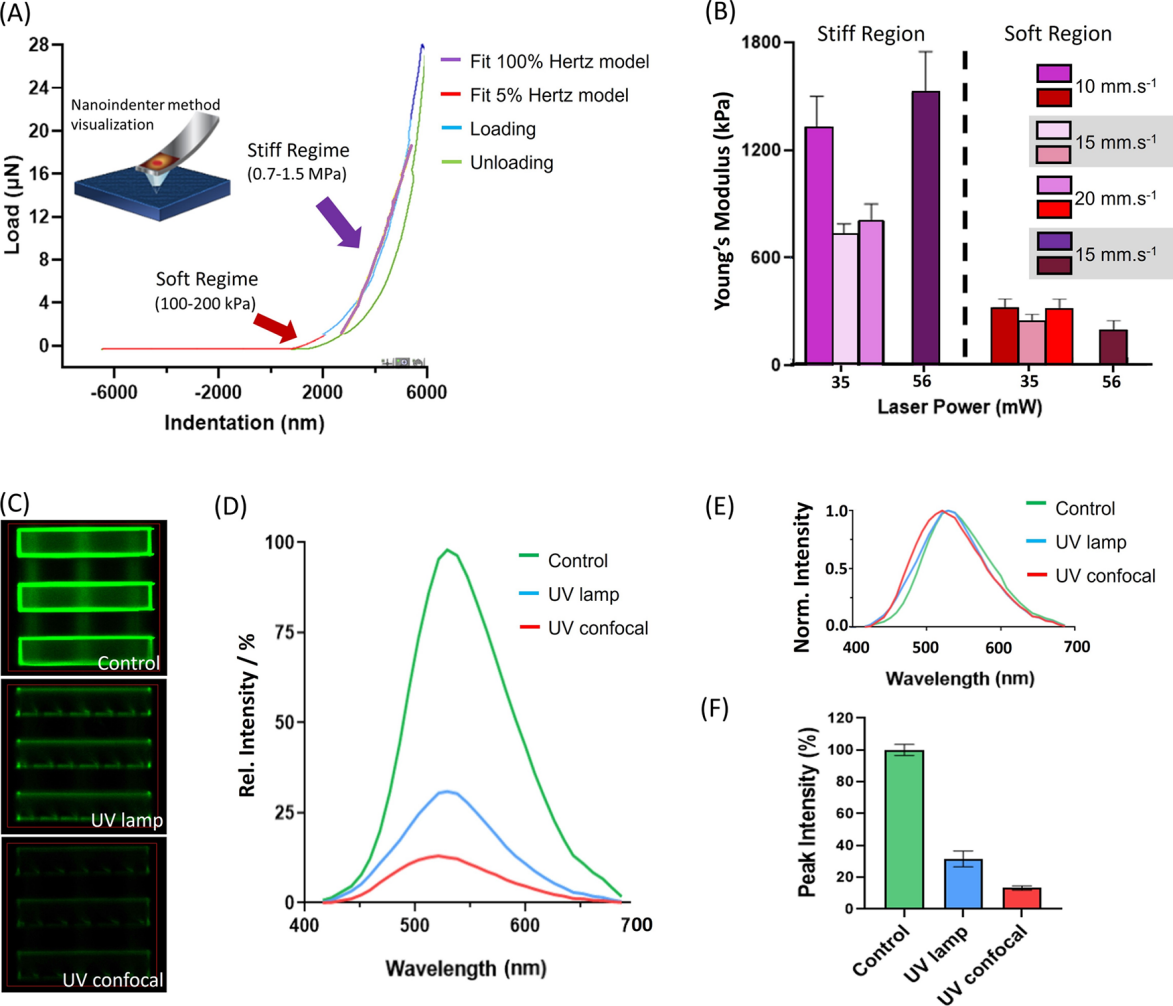
Characterization assays: (A) representative load-indentation curve with bilayer occurrence for a PEGDA 700 pedestal fabricated with *P*_L_ = 25 mW, *v*_WS_ = 10 mm·s^–1^, and 60 min water immersion time; (B) Young’s modulus of fabricated PEGDA 700 pedestals with different writing parameters fitted with 100% Hertz (stiff layer) and 5% Hertz (soft layer) models; (C) confocal images of different treated SZ2080 structures, focused on the bottom layers’ interface: control, UV lamp, and UV confocal; (D) fluorescence emission spectra plot dependence of the sample treatments collected over the area indicated by a red square; (E) normalized emission spectra; (F) bar diagram of peak (529 nm) intensity percentage with the control (no UV treatment) corresponding to 100%.

Each tissue has a specific stiffness intrinsically associated to its function and exposure to mechanical loading. The BM presents a Young’s modulus between 0.5 and 1.5 kPa.^18^ Material stiffness can greatly influence the differentiation of multipotent cells, justifying the need to study different alternatives.^45,46^ Unlike the stiffness of the commercial IP-S (*E* = 4.6 GPa) and SZ2080 (*E* = 2.8 GPa) polymers,^27,47^ the study of the mechanical properties of two-photon polymerized PEGDA 700 is still lacking in the literature, and therefore, its Young’s modulus was analyzed using a nanoindenting approach. Figure 2A shows the load indentation curve from a representative PEGDA 700 pedestal fabricated using the commercial 2PP *Nanoscribe* setup with two different fittings, 5 and 100% of the loading curve using the Hertzian model.^48^ The results reveal Young’s modulus heterogeneities throughout the structure, depending on the direct contact of the surface layers with water compared to the inner core or the amount of laser power/writing speed. As clearly observed by the two different slopes, we report considerably different Young’s moduli: a softer outer layer of approximately 2 μm thickness, featuring an average Young’s modulus of 250 kPa and a stiffer inner layer, with an average Young’s modulus of 1.1 MPa. The literature reports this range of values for PEGDA hydrogels but never mentioning this type of behavior.^49−51^ The described phenomenon may be induced by the overexposure of the inner layer, which is fabricated first. Furthermore, the hydrogel external layer is in direct contact with water, which likely increases its softness. The Young’s modulus values shown in Figure 2B relate to pedestals fabricated with different writing parameters. Higher laser powers seem to increase the outer layer stiffness, which most likely relates to a higher rate of polymerization. Lower writing speeds lead to longer local exposures, increasing the stiffness. The apparent independence of Young’s modulus values of the “soft region” shown in Figure 2B in relationship with the fabrication parameters can be potentially related to the interaction between the outer hydrogel layers and water before nano-indentation. The water may impact the hydrogel surface stiffness in a way that it decreases the fluctuations caused by different writing parameters. Eventually, some external factors during different fabrication sessions (such as room temperature, humidity, and slight developer concentration variations) may also influence this behavior. Considering the results, PEGDA 700 is a very attractive material for future in-depth studies on how stiffness affects the cell behavior, allowing shifting stiffness without changing the material itself.

### SZ2080 Autofluorescence Reduction

Even though SZ2080’s biocompatibility is well known,^17,52^ its bright autofluorescence often hinders the cellular interaction analysis, as the broadband fluorescent emission overlaps with most fluorescence markers. We find that the autofluorescence of SZ2080 structures can be efficiently reduced using a UV-light exposure of 2 h. Figure 2C shows the fluorescence microscopy images of the control, the UV lamp-treated, and the UV confocal-treated scaffolds. Figure 2D,E shows the respective emission spectra in relative (percentage) and maximum-normalized units. The UV-confocal and UV-lamp treatment methods achieve autofluorescence reductions of up to 90 and 70%, respectively. Previously reported chemical treatments, such as Sudan Black B rinsing,^52^ could be used in addition to the UV treatments to quench the fluorescence intensity further.

### Cell—3D Microstructure Interactions and Biocompatibility

**Figure 3:**
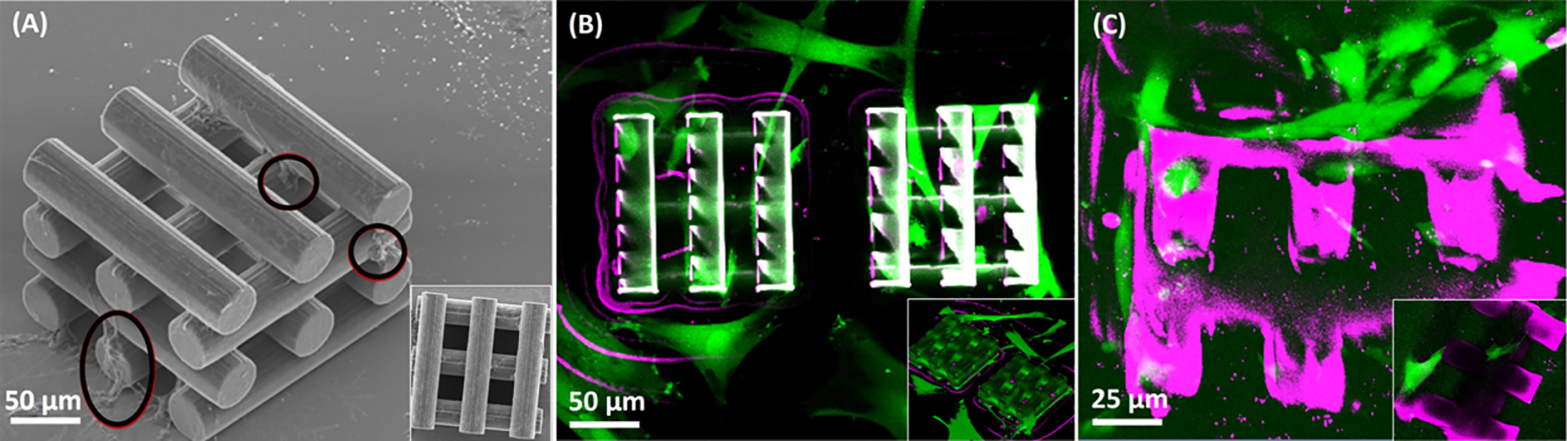
Representative images of BM-MSCs interacting on fibronectin-functionalized woodpile scaffolds with a gap of 25 μm after 6 days. (A) SEM images of BM-MSCs interacting on IP-S scaffolds. The black circles indicate places where BM-MSCs were attached prior to the fixing protocol. The inset shows the scaffold top view. (B) Bottom view confocal images of BM-MSCs interacting with SZ2080 structures functionalized with fibronectin taken with a 20× microscope objective. The inset shows the scaffold *z*-stack 3D projection 45° tilted (green—calcein-AM: live cells/SZ2080 material, magenta—EthD-1: dead cells). (C) Bottom view confocal images of BM-MSCs interacting with PEGDA 700 structures functionalized with fibronectin taken with a 20× microscope objective (green—calcein-AM: live cells, magenta—EthD-1: dead cells/PEGDA 700 material). The inset shows the scaffold lateral view with a cell stretching from one beam to another. In the confocal images, the objective is focused on the glass–structure interface, and structures are imaged through the glass and medium.

To study the structures’ biocompatibility, scaffolds of each material, with a fixed gap of 25 μm, and functionalized with fibronectin, were tested in the presence of BM-MSCs over 6 days (Figure 3).

The biocompatibility of IP-S woodpile scaffolds with BM-MSCs for the first 2 days out of a total of 6 assay days is observed with optical microscopy. BM-MSCs appear to be alive and have long extensions with the IP-S scaffolds for the first 2 days (optical microscope data shown in Figure S5). SEM images (Figure 3A) reveal multiple cell-IP-S interactions outside and inside the scaffold, with adhesion to both flat (end facet of the beams) and round scaffold surfaces, also in the upper layers. The inset in Figure 3A shows the top view. The IP-S samples were imaged using SEM due to the thickness of the glass substrate being incompatible with the focal distance of the confocal microscope objectives.

By analyzing the BM-MSCs’ interactions with SZ2080 scaffolds—Figure 3B—using confocal microscopy, we observe cells with long extensions and a low dead cell count (magenta). Adhesion to the upper and inner scaffold layers is detected from day one. In terms of material functionalization, we also tried fetal bovine serum (FBS). However, for the SZ2080 polymer, the results indicate that such a functionalization is not viable due to the generation of BM-MSC agglomerates in the scaffold, forming a dense tissue, where the majority would later die after 48 h due to their contact-inhibited feature (Figure S6). Contact inhibition is the abrupt arrest of the cell cycle when cells contact each other, ceasing growth, inducing slower proliferation, and reducing the differentiation capacity. Instead, functionalization with fibronectin allowed a progressive bottom/surface up growth, improving BM-MSCs’ interaction with 3D microstructures.

PEGDA 700 scaffolds after 6 days of BM-MSC incubation show adhesion in diverse places of the scaffold. In the confocal images taken at a selected image plane (Figure 3C), PEGDA 700 scaffolds with 6 days’ incubated BM-MSCs have shown adhesion in different regions. Further, in the inset of Figure 3C, several elongated live cells (green) are attached to the PEGDA 700 scaffold (magenta). Future studies should employ another color range for the identification of dead cells (e.g., EthD-1 with a blue fluorescence spectrum) to easily discriminate the PEGDA scaffold and dead cells. Both biocompatibility indicators, shape and calcein-AM fluorescence emission, suggest the presence of healthy cells. Cells’ spreading and growth were observed inside and around the scaffold, providing support for vertical growth. Cells partly stretch in elongated shapes, connecting one scaffold beam to another, thereby spanning 25 μm and more (Figure 3C—inset). The PEGDA 700 scaffold suffered delamination issues from the substrate; nonetheless, it did not compromise BM-MSCs–scaffold interactions. These scaffold–substrate adhesion problems when in contact with cell culture reagents demand further optimization procedures, for example, through new adhesion promoters. Notwithstanding, the conducted cell assays confirm that this material holds the potential to host BM-MSCs. Even though a blend of PEGDA has been used before to promote chondrogenic differentiation of MSCs, which proves this material’s potential,^53^ there are not yet studies in the literature about the biocompatibility of 3D PEGDA 700 scaffolds with BM-MSCs. Further, a limited number of studies mentioning the fabrication of PEGDA structures with 2PP can be pointed out, probably related to the challenges presented by this material in terms of mechanical stability and substrate adhesion. For the first time, we report the fabrication of complex 3D structures in PEGDA 700 and its interaction with BM-MSCs. Further approaches to increase the adhesion of hydrogel scaffolds in liquid media should be explored to increase the compatibility between PEGDA 700 studies and cell culture.

Overall, analyzing the cells’ morphology, it is observed that they maintain the typical elongated shape of a non-differentiated cell. This means that even with very distinct materials presenting higher stiffness than the original BM environments, cells could keep their multipotent feature. To support these first observations, the characterization of surface markers’ expression via flow cytometric analysis should be employed in a future study.

Concerning HeLa cells, they adhered, grew, and proliferated on the three materials, all excellent indicators of their suitability for cervical cancer cell models. We obtain excellent viability results, with the cells retaining their spindled-shaped morphology (Figures S7 and S8). IP-S scaffolds are biocompatible with HeLa cells for at least 6 days of culture, with evident growth in the *z*-direction and proliferation over time. This outcome is supported by previous cellular interaction studies where IP-resin scaffolds support the growth of HeLa cells.^33,54^ SZ2080 and PEGDA 700 scaffolds are biocompatible with HeLa cells for at least 3 days of culture. Since HeLa cells are one of the most viable and frequent cell lines used for initial tests, this parallel study reinforces the scaffolds’ versatility to host other cell lines than BM-MSCs.

### 3D Microstructure Gap Size Influence on the Colonization of BM-MSCs

**Figure 4:**
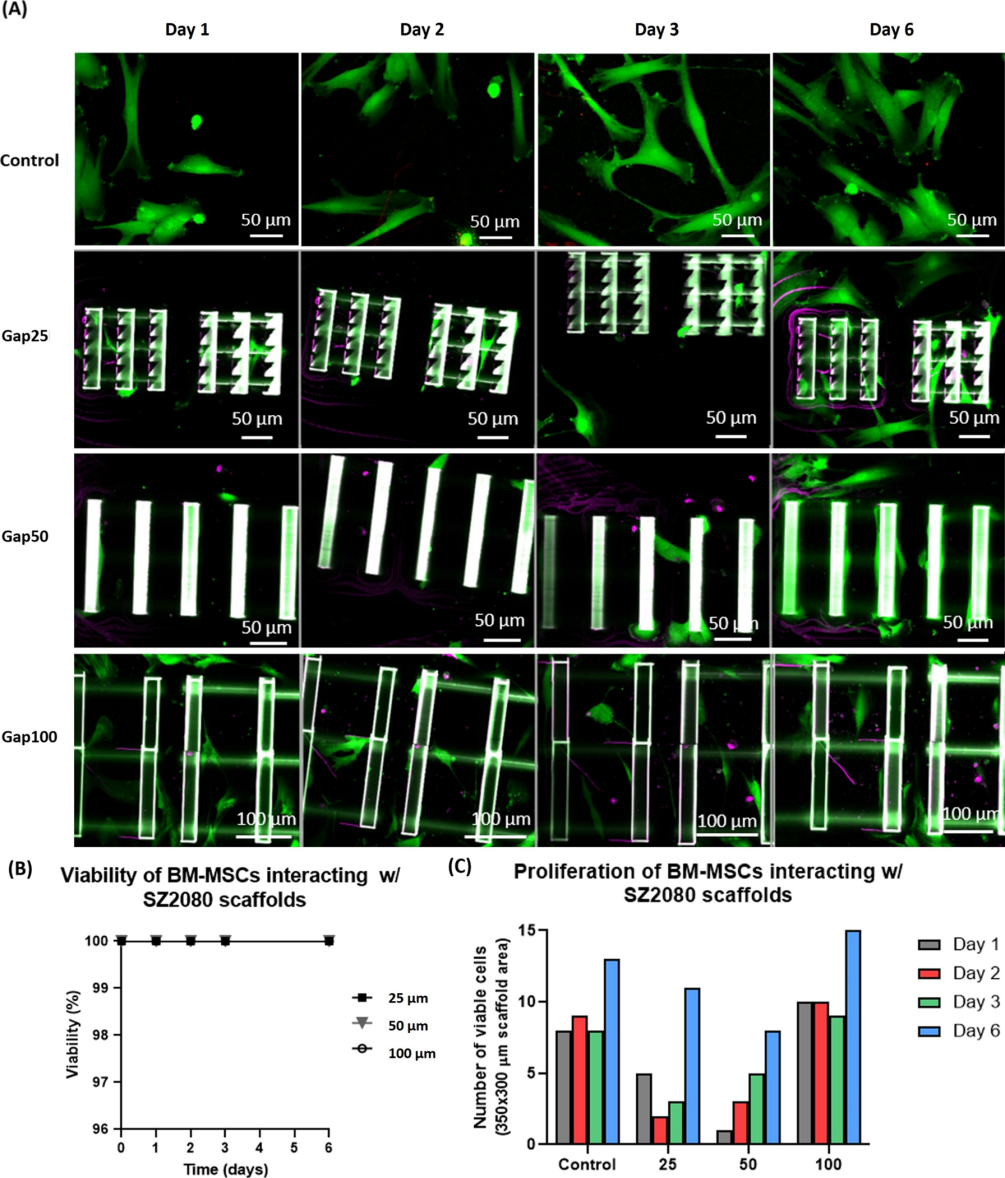
BM-MSCs’ interaction with SZ2080 woodpile scaffolds (gap 25, 50, and 100 μm) treated with fibronectin after 1, 2, 3, and 6 days: (A) bottom view confocal images, (B) viability analysis, and (C) proliferation analysis. The cell counting is performed in the total image area, bottom section (350 × 300 μm^2^). Scaffolds were functionalized with fibronectin. Green: live cells; magenta: dead cells; white: SZ2080 material; labels—number indicates the scaffold gap.

Figure 4A shows BM-MSC interactions with fibronectin-treated SZ2080 scaffolds featuring different gap sizes in a 2D plane. This material allows a consistent analysis due to its compatibility with confocal microscopy and mechanical stability. Considering both morphology and green staining of BM-MSCs, we can assess their state (dead or alive), count them, and perform statistical analysis. Overall, we find that the migration increased in the presence of larger gap sizes, with a minimal number of cells inside 25 μm gap scaffolds, which are smaller than the maximum 31 μm cell diameter.^41^ While previous works have reported good results with 20 μm pores, we found that cells struggle to migrate in that range of gap-size (25 μm).^32^ This difference may be justified by the different cell line used, namely, primary rat MSCs. Hence, 25 μm gap scaffolds shall be excluded from further studies in this paper. The 50 and 100 μm gap scaffold homed BM-MSCs in numbers that increase with the gap size.

### Cells’ Viability and Proliferation Analysis of BM-MSCs in Interaction with 3D Microstructures

Fluorescence microscopy was employed to analyze the cell viability instead of standard 2D viability and proliferation assays such as MTT, MTS, BrdU, or CCK-8, which are less suitable in the presence of 3D cell cultures concentrated in small volume fractions. Figure 4B shows a viability percentage of 100% until 6 days for all SZ2080 scaffolds treated with fibronectin, indicating a high level of biocompatibility for at least that period. The observed magenta points are not accompanied by green fluorescence and therefore are not related to dead cells. The magenta fluorescence is likely related to residues of other dead cells or polymer residues. The proliferation analysis shown in Figure 4C indicates a clear tendency to increase over time. The scaffold with a 100 μm gap shows the highest number of viable cells. Further, this scaffold has a higher proliferation rate than the other ones (25 and 50 μm gap). The behavior of the larger scaffold gap is very similar to the flat glass surface control.

We observe mostly living cells in PEGDA 700 scaffolds, supporting the material’s biocompatibility for at least 6 days, despite the scaffold instability, which made high-resolution imaging over a longer time difficult (see Figure S9). Further studies should be conducted to confirm the observed high viability rates. The overall scaffold size does not seem to play a fundamental role in the cells’ viability. As previously mentioned, the viability of IP-S scaffolds was not assessed with fluorescence assays. Nevertheless, BM-MSCs with a cell morphology indicating healthy or live cells were found on IP-S scaffolds after 2 days of seeding, using an optical microscope.

## Conclusions

In the current study, we optimized reproducible 3D woodpile structures with a 25 μm beam diameter and 25, 50, and 100 μm gaps using 2PP. These structures were fabricated with three different materials, IP-S, PEGDA 700, and SZ2080, and we assessed their interaction with BM-MSCs and HeLa cells.

For the PEGDA 700 material, we are not aware of any published results combining the 2PP technique and BM-MSCs. Previous works have reported on the biocompatibility of IP-resins, SZ2080, and PEGDA 700 hydrogel 3D microstructures. However, the essays were conducted using different fabrication setups, other 3D geometries or other cell lines.

For the first time, we observed by using nanoindentation a complex Young’s module profile in the custom-formulated PEGDA 700 material. Interestingly, two different stiffness regions could be distinguished between the inner core of the hydrogel structure (stiff: 0.7–1.5 MPa Young’s modulus) and the outer core (soft: 100–200 kPa Young’s modulus), which may be associated to water solvent exposure during immersion in the aqueous environment and/or laser overexposure during fabrication. These findings provide novel opportunities for cell–interface interaction studies preserving the same material while modifying the interface material’s stiffness.

We also present a new protocol to reduce the SZ2080 autofluorescence and allow multistaining live–cell interaction studies. Previous studies report that the SZ2080 fluorescence decreases only around 50% through chemical treatments, while we achieved an almost 90% reduction with focused UV illumination.

All woodpile structures showed biocompatibility and promising capabilities to home different cell lines. Indeed, the fabricated 3D scaffolds are biocompatible, and proliferation levels are similar to those found in 2D, a starting point for new future work and the in-depth study of in vitro disease models or differentiation processes. We found that fibronectin functionalization significantly improves the BM-MSC–scaffold interaction quality compared to FBS. Focusing on the cells’ morphology when interacting with the microscaffolds, more elongated shapes can be seen either along the individual polymer structures or even spanning up to 50 μm to reach different scaffold elements, indicating a high affinity. Comparing the three different-dimensioned 3D woodpile scaffolds for the homing of BM-MSCs, the 100 μm gap scaffolds presented the most promising results with higher homing rates than the smaller 50 and 25 μm gap sizes. The scaffolds with a 25 μm gap hamper the migration of BM-MSCs to the scaffold core, probably because BM-MSCs are too large to migrate into those pores.

Similar results were found in the complementary studies with HeLa cells, which corroborates the potential of the 3D woodpile scaffolds for growing different cells lines. HeLa cells’ penetration within 25 μm gaps is reduced compared to other gap sizes but not entirely prevented. This supports the idea that the woodpile scaffold gap strongly influences cell migration, and if the gap size is close to the cell size, it prevents the cells from migrating and adhering inside the 3D structures.

Our findings reported the use of 2PP in the presence of different polymeric and hydrogel materials for the generation of complex 3D structures. The structures’ dimensions were optimized for cells with diameters of 20–30 μm. However, they can be easily tuned and adapted for other cell lines, considering that migration is favored by pores thrice the size of the cells’ diameter. Recreating complex 3D microenvironments where cells can adhere and grow paves the way for drug testing and in-depth studies of cellular behaviors. Future studies will include the investigation of the effect of hydrogel stiffness changes on BM-MSCs’ morphology and differentiation processes, requiring thus longer cell culture times up to several weeks. Interesting morphologic changes were observed for cells in direct contact with the scaffold, which deserve further studies relating the observed morphologies with those found in the natural BM tissue.

Scaffolds may provide promising platforms in medical and pharmaceutical research, where 3D cellular models are used for disease modeling applications or as drug screening tools, which show improved reproducibility of drug responses and cell morphology or enzymatic functions.^55−57^ Several challenges lay ahead for the development of scaffolds for TE, including the generation of dense cellular assemblies, either by increasing incubation times or the use of already formed 3D cell spheroids. From the perspective of enabling new functionalities, particles or chemicals could be added to the polymer to improve the stability, add new active features, and lead to the realization of “smart” scaffolds. To employ these 3D scaffolds for developing models of leukemic diseases, future studies are required to assess the multipotent characteristics of the BM-MSCs in a 3D spatial configuration as well as to test the co-culturing with relevant cell lines, such as hematopoietic stem cells, and specific ECM components, for example, collagen I and hydroxyapatite. Further, to use these platforms as a starting 3D BM model for disease and therapeutic studies, the prolonged incubation times can rely on the use of microfluidics-based approaches for automated and controlled nutrient delivery.

## Experimental Section

### Materials

IP-DIP is a commercial negative-tone resist (Nanoscribe) for high-resolution 3D printing compatible with the 63× NA 1.4 objective and DiLL configuration. The cleaning procedure of the fused silica substrates (*Nanoscribe*, 25 × 25 mm^2^ and thickness 0.7 mm) is performed with acetone and isopropyl alcohol (IPA), followed by an oxygen plasma treatment (OPT). The OPT procedure is conducted using a plasma system (Femto model, 30 W, 200 mbar, 5 min, *Diener Electronic*). The basic chemical development of this material, employed to remove the unexposed photoresin, consists in 25 min of immersion in PGMEA (*Sigma-Aldrich*) and a 5 min rinse with IPA. A combination of PGMEA and Novec (*Sigma-Aldrich*, 7100 Engineered Fluid) was evaluated to decrease development times and surface tensions. The IP-DIP material is very similar to IP-S and therefore was only used for optimization purposes and not for the final scaffolds for cell interaction studies. A total of 16 printing parameter combinations were tested, four different laser powers (*P*_L_ ranging from 25 to 40 mW with a step size of 5 mW), and four writing speeds (*v*_ws_ ranging from 10 to 25 mm·s^–1^ with a step size of 5 mm·s^–1^).

IP-S is a liquid negative-tone methacrylate photopolymer employed in the *Nanoscribe* setup with a 25× NA 0.8 objective and DiLL configuration. The employed substrates are indium tin oxide (ITO)-coated glass substrates recommended for the 25× objective (Nanoscribe, 25 × 25 mm^2^ and thickness 0.7 mm). The ITO coating facilitates the detection of the interface between the photosensitive polymer and the substrate. The cleaning procedure is the same of IP-DIP (acetone, IPA, and OPT 30 W, 200 mbar, 5 min). The optimized structures are obtained with 100 mm s^–1^ and the maximum *P*_L_ (50 mW). The slicing and hatching distances are kept constant at 0.4/0.4 μm. The development to remove the unexposed resist consists of 15 min with PGMEA followed by 30 s of rising with Novec.

The SZ2080 acquired from *IESL-FORTH* is a photosensitive hybrid polymer material with a low shrinking behavior as well as stable mechanical and chemical properties. The glass substrate (*Fischer Scientific*, 50 × 24 mm^2^ and 0.17 mm thickness) is first cleaned with acetone, IPA, and a nitrogen pistol. Then, it is heated at 95 °C for 20 min (hot plate VMS-C7 Advanced, *VWR*). The following step is drop-casting 40 μL of SZ2080 with a micropipette on the glass substrate as much centered as possible. A new heating procedure is conducted at 95 °C for 30 min to eliminate air bubbles, evaporate the solvent, and improve the contact between the glass substrate and the material. The selected objective is the 40× NA 0.75. For the development phase, 5 mL of 4-methyl-2-pentatone (*Sigma-Aldrich*) and 10 mL of IPA are mixed in a beaker.

PEGDA is a hydrogel not intrinsically sensitive to light exposure. To have a photosensitive material compatible with 2PP, PEGDA 700 (*Sigma-Aldrich*) was mixed with the photoinitiator phenylbis(2,4,6- trimethylbenzoyl)phosphine oxide (*Sigma-Aldrich*), also known as IRGACURE 819. For a 1% weight concentration of the photoinitiator, 0.05 mg of IRGACURE 819 was mixed with 5 mL of the PEGDA hydrogel in a dark glass vial using a magnetic stirrer for 2 h and then stored at 7 °C. The photosensitive hydrogel vial is taken from the fridge around 2 h before the printing session. The material was printed with a 20× objective on a glass substrate (*Fischer Scientific*, 50 × 24 × 0.17 mm^3^). Further, a pretreatment is conducted on the glass slide to promote the material adhesion. The treatment consists in 2 h of immersion within a 0.5% v/v 3-(trimethoxysilyl)propyl methacrylate (MAPTMS, *Sigma-Aldrich*)/ethanol (99.8%) solution. The PEGDA material is then drop-casted and heated on a hot plate (VMS-C7 Advanced model, *VWR*).

Table 1 presents the different processing parameters used for each 2PP setup to obtain optimized structures for the respective materials under study.

**Table 1:** Summary of the Processing Parameters Used for Each 2PP Setup to Obtain Optimized Woodpile Structures for the Respective Materials Under Study^a^

material	IP-S	SZ2080	PEGDA 700
setup	photonic professional GT2, *Nanoscribe*	custom 2PP setup	custom 2PP setup
design software	AutoCAD	MATLAB	MATLAB
power	50 mW	20 mW	20 mW
writing speed	100 mm s^–1^	200 μm s^–1^	200 μm s^–1^
slicing/hatching	0.4/0.4 μm	0.5/0.2 μm	0.5/0.2 μm
mode	dip-in (laser-polymer-substrate)	air (laser–substrate–polymer)	air (laser–substrate–polymer)
glass substrate (*l* × *w* × *h* mm^3^)	25 × 25 × 0.7 ITO coated	24 × 50 × 0.17	24 × 50 × 0.17
objective	25× NA 0.8	40× NA 0.75	20× NA 0.45
printing times	25 μm|6.9 min	25 μm|33 min	25 μm|30 min
	50 μm|9.3 min	50 μm|1 h	50 μm|55 min
	100 μm|14.6 min	100 μm|1 h 27 min	100 μm|1 h 20 min
substrate preparation	cleaning (acetone and IPA), OPT (30 W, 200 mbar, 5 min) and drop cast	cleaning with acetone/IPA, heated at 100 °C for 20 min, drop cast and 30 min heated at 95 °C	cleaning (acetone/IPA), OPT (30 W, 200 mbar, 5 min) and 2 h silane treatment with MAPTMS/ethanol; rinse with DIW, drop cast and 30 min heated at 95 °C
development	15 min PGMEA/30 s Novec rinse	2 h in 4-methyl-2- pentatone/IPA, 5 min ethanol, 2 h UV	40 min ethanol

a MAPTMS—3-(trimethoxysilyl) propyl methacrylate, DIW—deionized water, IPA—isopropanol, PGMEA—propylene glycol methyl ether acetate, OPT—oxygen plasma treatment.

### Scaffolds’ Design and Fabrication Parameters

Prior to the scaffold’s fabrication, a challenging architecture-denominated microgrid is fabricated with the IP-DIP material to optimize several printing parameters and development procedures (see the Supporting Information). The microgrid has round beams with a 1 μm diameter spaced 9 μm apart. The cell scaffolds’ design is based on a 3D micron-scale woodpile architecture, with a total of four layers. This structure is attractive because the cell proliferation is affected by the interconnected channels and by the stack of beams.^58,59^ The beam diameter was kept fix at 25 μm, while the beams’ spacing was varied between 25, 50, and 100 μm. For the development of 3D scaffolds, three different materials were used, namely, IP-S, SZ2080, and PEGDA 700. The design was also studied through simulations prior to fabrication to understand the correction factors capable of minimizing oversized structures caused by an increased voxel.^43^

### *Nanoscribe* Photonic Professional GT2 Used for IP-S Material

The 3D IP-S structures were fabricated with 2PP using a commercial microfabrication device (GT+, *Nanoscribe*) and a femtosecond-pulsed (100 fs, 50 mW) fiber laser (FemtoFiber Pro, *Toptica Photonics*) beam at 780 nm. In the “galvo” configuration, galvanometric mirrors laterally scan the laser beam, and the vertical movement is controlled by piezoactuators, which allows a fast fabrication process. The structures are designed using the software *Autodesk Inventor Professional 2019* and transferred to DeScribe (*Nanoscribe* software) which applied the desired slicing/hatching parameters to the design prior to printing. A 25× NA 0.8 objective (*Zeiss*) was employed with the IP-S material. In the DiLL configuration, the objective is directly in contact with the photosensitive resist, which minimizes spherical aberrations.

### Custom-Inverted 2PP Setup Used for SZ2080 and PEGDA 700 Materials

A 2PP custom-designed setup was used to fabricate PEGDA 700 and SZ2080 structures. The setup comprises a femtosecond pulsed titanium Sapphire-based laser (Tsunami 3960C-15HP, *Spectra-Physics*) operating at a repetition rate of 80 MHz, tuned to 780 nm for 2PP of both materials. It includes a pump laser (Millenia 15, *Spectra Physics*) focused onto a Ti:Sapphire crystal in a femtosecond laser cavity. The laser output power of around 1.5 W is attenuated on the optical path to an inverted microscope setup (RM21, *Mad City Labs*), equipped with an *XY* microscanner (MicroStage, *Mad City Labs*) and an *XYZ* piezo-nanopositioning system (NanoLPS200, *Mad City Labs*). A microcontroller (DFRduino UNO V3.0, *DFRobot*) and custom-developed device control software with graphical user interface and panels (Python) are used for alignment and fabrication control. The microcontroller communicates with a shutter controller. On the excitation path, optical components are used for power adjustment (gradient attenuator wheel NDC-50C-2M, *Thorlabs*), and to overfill the back aperture of the objective, a beam expander (double lens *f*_BE1_ = 40 mm and *f*_BE2_ = 150 mm, B AR-coated, *Thorlabs*) is used. The beam steering takes place using silver mirrors (PF10-03-P01-10, *Thorlabs*) along the beam path. The main components responsible for the power attenuation are the lambda half-plate, prisms, neutral-density (ND) filter, and also during the over-illumination of the back aperture.

Some of the fundamental components for the setup’s functioning are the beam splitter and the objective, chosen according to the final application. The selected option was using a 0.2 ND filter. For focusing and placing the sample, a normal lamp is covered with UV-protective foil to avoid one-photon excitation of the polymer, while for transmission imaging, a camera was employed (MCE-B013-UW, *Mightex*). The laser can largely be blocked by placing a BG39 filter in front of the camera. The experiments are carried out with air objectives, a 20× objective (CFI S Plan Fluor ELWD, 0.45 NA, *Nikon*) for PEGDA 700 and a 40× objective (Nikon Plan Fluorite Imaging Objective, 0.75 NA, *Thorlabs*) for the SZ2080 material. The structures are designed using a developed *MATLAB* (2018v2) script that produces a .gcode file as the output.

### Morphological Properties’ Characterization Using SEM

IP-resin samples were imaged using a scanning electron microscope (model JSM-6010LA, *Jeol*) after sputtering the samples with approximately 12 nm thick gold, using a sputter coater (JFC-1300, *Jeol*). Images and measurements are taken from the top of the architecture and 45° tilting to thoroughly characterize the structure. For the SZ20080 and PEGDA 700 materials, a second SEM setup (QUANTA 650FEG, *FEI Europe B.V.*) is employed. The second sputtering system is an ultra—HV multitarget confocal sputtering tool (*Kenosistec*), used for depositing 10 nm of gold (49 s). Images are taken under HV with 10 kV and maximum or no tilting.

Before performing SEM characterization of cell–scaffold interactions, it is necessary to fix and dehydrate the cells. This protocol follows the one described in Accardo et al.^34^ First, the cells are washed with phosphate buffered saline (PBS) and incubated in a 4% formaldehyde solution for 4 h at room temperature (RT). Then, the fixing solution is removed, and the cells are washed with PBS. After this, cells are incubated in 50, 70, 90, and 100% ethanol for 4 min in each step and air-dried at RT.

### Mechanical Properties’ Characterization Using Nanoindentation

A Piuma nanoindenter (*Optics 11*) is used to evaluate the hydrogel stiffness. It is suited for soft materials and measurements within physiological conditions (sample immersed in a liquid environment). It covers a wide range of measurable Young’s moduli *E* (between 1 Pa and 1 GPa). Unlike the hydrogel, the stiffness of IP-resins and SZ2080 can reach values from 2.8 to 4.6 GPa, which makes these materials incompatible with the equipment. Further, the mechanical properties of the commercially available IP-resins and SZ2080 materials are widely analyzed in literature contrarily to PEGDA 700.^27,47^ From the built-in mechanical models, it is possible to extract the effective Young’s modulus *E*_eff_. After the mechanical assay, the data analysis can be performed with the software *DataViewer*. For the mechanical characterization, PEGDA 700 pedestals 100 (*x*) × 100 (*y*) × 50 (*z*) μm^3^ were printed, with different writing parameters. After the developing process, the samples remain in water. With a tip radius of 28 μm and pedestals 50 μm thick, indentations can reach approximately 4.5 μm maximum depth (16% of tip radius), which defines a circle with a 9 μm diameter. For this reason, within a single scan, four indentations are performed, 40 μm apart, forming a square. For each indentation, the *E*_eff_ is extracted using the Hertz model (the most suitable model for soft materials). The stiffness of the glass tip is 4.2 N·m^–1^ (measurable nominal Young’s modulus range ≈ 10 kPa to 10 MPa).

### Autofluorescence Reduction and Characterization for the SZ2080 Material in a Confocal Microscope Setup

SZ2080 is a polymer material characterized by a high autofluorescence in a wide range of wavelengths, which causes cell camouflage and strongly impairs the image quality using fluorescence assays. Since the literature mention UV treatments as viable option to quench fluorescence,^60^ this work employs two different UV sources. A UV lamp (M365LP1-C1 collimated LED, *Thorlabs*), with a nominal wavelength of 365 ± 9 nm, is placed inside a dark box, approximately 1 cm distance from the sample. The second UV treatment is performed inside a confocal microscope (LSM780, *Zeiss*), where the light from a halogen lamp is filtered with a 365 ± 15 nm bandpass filter (DAPI filter) and focused with a 20× microscope objective onto the polymer sample. Both treatments have the duration of 2 h. A sample without UV illumination was used as the control. This assay resorts to a confocal microscope (LSM780, *Zeiss*) used in a lambda model and with a laser excitation at 405 nm at 0.32 mW.

### Cell Culture and Cell Plating for Microscopy

A human BM-MSC (*LGC Standards*) culture is performed following the Biological *Industries*’ protocol for passaging MSCs. Briefly, BM-MSCs are cultured in a cell medium composed by an MSC basal medium (PCS500030, *ATCC*) and an MSC Growth Kit (PCS500041, 35 mL of FBS, 0.5 mL of rh IGF-1, 0.5 mL of Rh FGF-b, 6 mL of l-alanyl-l-glutamine, *ATCC*). The cell culture medium is replaced every 2 days. When the cells are 70% confluent and for a T75 flask, the old medium is removed and 10 mL of PBS (1×, 21-040-CV, *Corning*) is added to wash the culture surface. To detach the BM-MSCs, PBS is replaced by 2 mL of warm trypsin–ethylenediaminetetraacetic acid (EDTA, 1× 0.25%, 25-053-CI, *Corning*), and the cells are incubated at 37 °C for 5 min. After that period, trypsin is diluted with 8 mL of pre-warmed complete medium. The cell suspension is centrifuged at 200*g* for 5 min, the supernatant is removed, and the cell pellet is re-suspended in 5 mL of warm medium. 1:5 of the total amount of cells is added to a flask with 12 mL of warm medium.

The culture process of HeLa cells follows the Fundamental Techniques’ recommendations in Cell Culture, Laboratory Handbook 3rd edition, from *Sigma-Aldrich*.^61^ HeLa cells provided by the Ultrafast Bio and Nanobiophotonics group (INL) are cultured in growth medium composed by the minimum essential medium (MEM, w/phenol red, L0416-500, *Biowest*) and supplemented with 10% FBS (HyClone FetalClone III, SH30109.03, *GE Health Life Sciences*) and 1% penicillin/streptomycin (P06-07100, *PAN Biotech*) at 37 °C and with 5% CO_2_. The cell culture medium is changed every 2 days. When the cells reach 80% of confluence and for a T75 flask, the cells are washed three times with 5 mL of warm PBS (1×, 21-040-CV, *Corning*) followed by incubation of 2 mL of warm trypsin–EDTA (1× 0.25%, 25-053-CI, *Corning*) for about 5 to 7 min. Then, 9 mL of warm medium is added to inactivate trypsin, and 90% of the cell suspension is discarded and replaced with fresh growth medium.

To perform sterilization, the scaffold glass substrate is first washed iteratively with ethanol and miliQ water three times each. The scaffolds are immediately sterilized for 1 h under UV light. After sterilization, a two-well culture silicone insert (*Ibidi*) is placed over the scaffolds. Two different functionalization components were tried, namely, FBS and fibronectin (F0895, *Sigma-Aldrich*), both for 30 min. Fibronectin is an ECM common component, previously used to functionalize scaffolds for BM-MSC seeding.^62^ Following *Corning*’s recommendations, 15,000 cells are seeded in each well to fulfill the amount of 70,000 cells cm^–2^.

### Staining for Live/Dead Assay and Multicolor Confocal Imaging

The live/dead assay allows a quantitative analysis of viability at the time of staining through enzymatic activity. The selected reagents to perform this assay were calcein-AM (*Invitrogen*, Ex/Em = 494/517 nm), staining live cells green, and EthD-1 (*Sigma-Aldrich*, Ex/Em = 528/617 nm), staining dead cells magenta. The whole procedure, from stock solution preparation until the staining protocol, follows the *Thermo Fisher* LIVE/DEAD viability/cytotoxicity kit for mammalian cells protocol. The resulting solution contains approximately 2 μM of calcein-AM and 4 μM of EthD-1. Materials are considered biocompatible for viability percentages over 70%, according to the criteria of the ISO10993-5 standard.^63^ To perform this assay, a multicolor confocal microscope (*Zeiss, LSM780*) is used. This imaging is not performed on IP-S samples since the glass substrate is too thick to image with the confocal microscope.

## Supporting Information Available

SEM images of a 3D microgrid; schematic of fabrication configuration and custom setup; MATLAB simulations; microscopy images of BM-MSCs interacting on IP-S scaffolds; confocal images of BM-MSCs interacting with FBS-functionalized SZ2080 scaffolds; microscopy images of HeLa cells interacting with woodpile scaffolds, viability, and proliferation assays; and BM-MSCs interacting with PEGDA 700 scaffolds (PDF)

## Abbreviations

2D: two-dimensional
2PP: two-photon polymerization
3D: three-dimensional
AM: addictive manufacturing
BM: bone marrow
DILL: dip-in laser lithography
DIW: deionized water
DLW: direct laser-writing
ECM: extracellular matrix
EDTA: ethylenediaminetetraacetic acid
FBS: fetal bovine serum
Gel: gelatin
IPA: isopropanol
IRGACURE: phenylbis(2,4,6-trimethylbenzoyl)phosphine oxide
ITO: indium tin oxide
MAPTMS: 3-(trimethoxysilyl)propyl methacrylate
MEM: minimum essential medium
MSCs: mesenchymal stem cells
NA: numerical aperture
ND: neutral-density
OPT: oxygen plasma treatment
PBS: phosphate buffered saline
PEGDA: poly(ethylene glycol)diacrylate
PGMEA: propylene glycol methyl ether acetate
*P*_L_: laser power
RT: room temperature
SEM: scanning electron microscopy
TE: tissue engineering
*v*_WS_: writing speed
