# Correction to “Cellular Interaction of Bone Marrow Mesenchymal Stem Cells with Polymer and Hydrogel 3D Microscaffold Templates”

**DOI:** 10.1021/acsami.4c15976

**Published:** 2024-09-25

**Authors:** Beatriz N. L. Costa, Ricardo M. R. Adão, Christian Maibohm, Angelo Accardo, Vanessa F. Cardoso, Jana B. Nieder

**Affiliations:** † INL−International Iberian Nanotechnology Laboratory, Ultrafast Bio- and Nanophotonics Group, Av. Mestre José Veiga S/n, 4715-330 Braga, Portugal; ‡ CMEMS-UMinho, University of Minho, DEI, Campus de Azurém, Guimarães 4800-058, Portugal; § Faculty of Mechanical, Maritime, and Materials Engineering (3mE), Department of Precision and Microsystems Engineering (PME), Delft University of Technology, Mekelweg 2, Delft 2628 CD, The Netherlands; ∥ CF-UM-UP, Centro de Física das Universidades do Minho e Porto, Universidade do Minho, Campus de Gualtar, 4710-057 Braga, Portugal; ⊥ Escola de Enxeñaría de Minas e Enerxía, University of Vigo, 36310 Vigo, Pontevedra, Spain

In our original article, an affiliation is missing for the first author of the paper (Beatriz N. L. Costa). The correct affiliation information for Beatriz N. L. Costa is as follows: INL–International Iberian Nanotechnology Laboratory, Ultrafast Bio- and Nanophotonics Group, Av. Mestre José Veiga S/n 4715-330 Braga, Portugal; CMEMS-UMinho, University of Minho, DEI, Campus de Azurém, Guimarães 4800-058, Portugal; Faculty of Mechanical, Maritime, and Materials Engineering (3mE), Department of Precision and Microsystems Engineering (PME), Delft University of Technology, Mekelweg 2, Delft 2628 CD, The Netherlands; **Escola de Enxeñaría de Minas e Enerxía, University of Vigo, 36310 Vigo, Pontevedra, Spain**.

All other affiliations and the present address are the same as in the original article.

This addition of the affiliation does not alter the conclusions, results, or interpretations presented in the original article. We regret the omission of this affiliation and apologize for any confusion or inconvenience this may have caused.

