# Supplementary material for: Cellular Interaction of Bone Marrow Mesenchymal Stem Cells with Polymer and Hydrogel 3D Microscaffold Templates
Source: ACS Appl Mater Interfaces. 2022 Mar 13;14(11):13013–24. doi: 10.1021/acsami.1c23442 (PMC8949723; doi:10.1021/acsami.1c23442)
Supplement: Supplementary file 1 — am1c23442_si_001.pdf [file am1c23442_si_001.pdf]

Supporting Information

Cellular Interaction of Bone Marrow Mesenchymal Stem Cells with Polymer and Hydrogel 3D Microscaffold Templates

Beatriz N. L. Costa, [a,b,c] Ricardo M.R. Adão, [a] Christian Maibohm, [a] Angelo Accardo, [c,]* Vanessa F. Cardoso,[b,d,e]* and Jana B. Nieder. [a,]*

[a] *INL - International Iberian Nanotechnology Laboratory, Ultrafast Bio- and Nanophotonics group, Av. Mestre José Veiga s/n, 4715-330, Braga, Portugal*

[b] *CMEMS-UMinho, University of Minho, DEI, Campus de Azurém, Guimarães 4800-058, Portugal*

[c] *Delft University of Technology, Faculty of Mechanical, Maritime, and Materials Engineering (3ME), Department of Precision and Microsystems Engineering (PME), Mekelweg 2, Delft 2628 CD, The Netherlands*

[d] *CF-UM-UP, Centro de Física das Universidades do Minho e Porto, Universidade do Minho, Campus de Gualtar, 4710-057 Braga, Portugal*

[e] *LABBELS-Associate Laboratory, Braga, Guimarães, Portugal*

* corresponding authors: A.Accardo@tudelft.nl, vcardoso@cmems.uminho.pt, jana.nieder@inl.int

KEYWORDS: two-photon polymerization, three-dimensional scaffolds, woodpile structures, polymer, hydrogel, bone-marrow mesenchymal stem cells, tissue engineering

Optimization of the 2PP Fabrication Process. This optimization process provides essential feedback about the polymerization process and the effects of several parameters, e.g., writing speed, laser power, development techniques, and design settings. For low exposure conditions, visible deformations result in collapsed structures (e.g., P_L =25 mW, v_{WS} =25 mm.s^{-1}), or asymmetric piling up (e.g., P_L =35 mW, v_{WS} =20 mm.s^{-1}), sometimes strictly at the center of the structure (P_L =30 mW, v_{WS} =20 mm.s^{-1}). In the end, five sets of parameters were considered viable, namely P_L =35 mW/ v_{WS} =10-15 mm.s^{-1} and P_L =40 mW/ v_{WS} =10-20 mm.s^{-1}. It is proven that the mechanical stability can be improved by adding a thin polymerized layer underneath the primary structure, henceforth referred to as pedestal (Figure S2A). This approach is only suitable for DiLL configurations, otherwise, the pedestal impairs the laser path (Figure S3A). Furthermore, we observe that using the solvent Novec for IP-DIP reduces surface tensions and increases structure fidelity compared to IPA (see Figure S2B). All the inputs gathered at this stage in terms of writing parameters, pedestal addition, and development method based on the use of the IP-DIP material were fundamental to identifying writing parameters for the IP-S material, which has similar chemical properties. Both IP-DIP and IP-S belong to the same manufacturer (*Nanoscribe*), and they are very similar in chemical composition and mechanical properties. The results were fundamental to facilitate and accelerate the IP-S scaffolds fabrication and optimization.

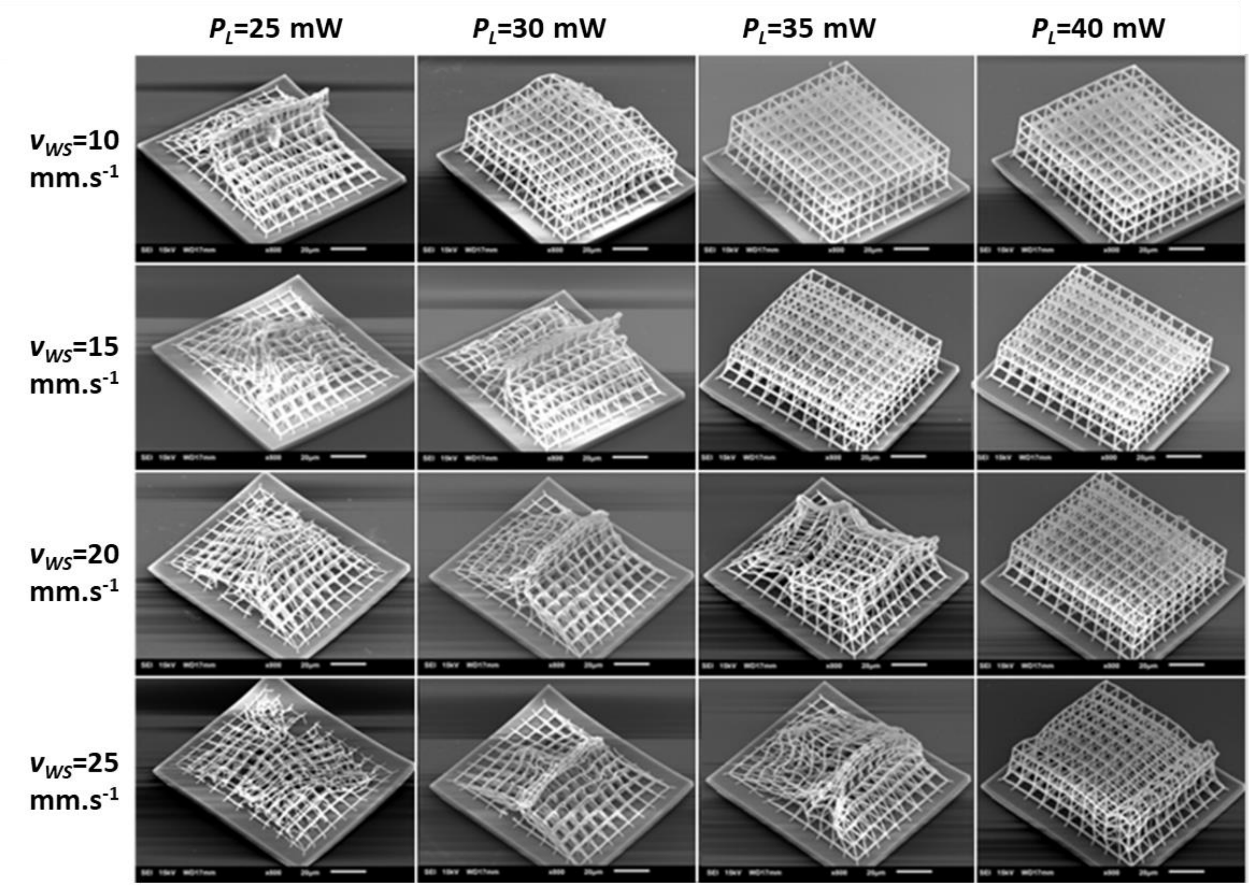

Figure S1. Representative SEM images of 3D IP-DIP microgrid with a 9 μm gap and 1 μm diameter with pedestal addition - P_L and v_{WS} influence. The structures were rinsed with IPA and printed on a fused silica substrate. Fabrication in DiLL mode using the Nanoscribe setup. P_L ranging from 25 to 40 mW and v_{WS} from 10 to 25 mm.s^{-1}. Images at 45°, 15 kV, x800 magnification, and a scale bar of 20 μm.

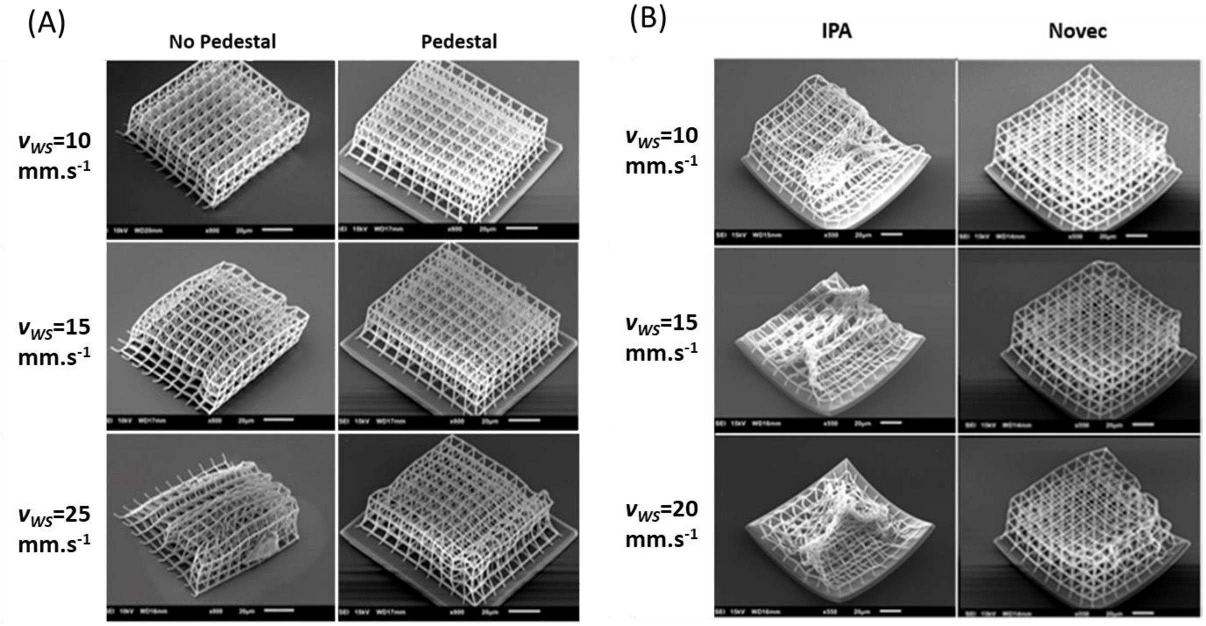

Figure S2. Representative SEM images of IP-DIP 3D microgrid with: (A) 9 μm gap and 1 μm diameter - pedestal (5 μm high) addition influence. Rinsed with IPA, obtain with DiLL mode, and printed on a fused silica substrate. P_L = 40 mW and v_{WS} ranging from 15 to 25 mm.s^{-1}; (B) 15 μm gap and 1 μm diameter with pedestal addition - development method influence. The structures were rinsed with IPA or Novec and printed on a fused silica substrate. Fabrication in DiLL mode using the Nanoscribe setup. P_L = 45 mW and v_{WS} from 10 to 20 mm.s^{-1}. Images at 45°, 15 kV, x800 magnification, and a scale bar of 20 μm.

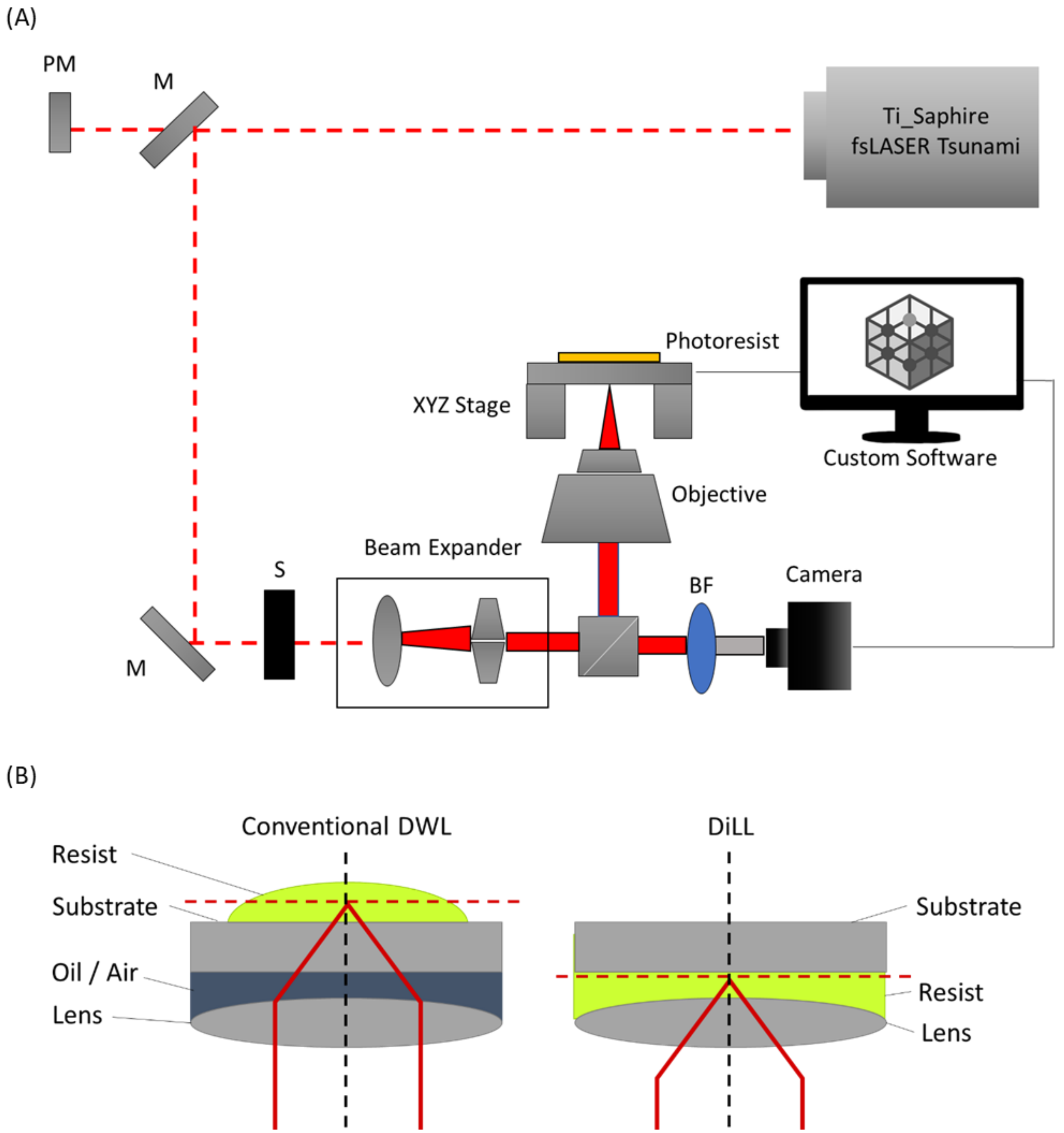

Figure S3. Simplified schematic representation of the: (A) custom-assembled inverted setup and its peripherals. PM – power meter, S - shutter, M - mirror, BF - blue filter; (B) differences between conventional or oil immersion mode (bottom-up fabrication) and DiLL mode, namely in terms of the positioning of the photoresist or another additional component in the substrate relatively to the lens.

Fabrication of the 3D Woodpile Structures.

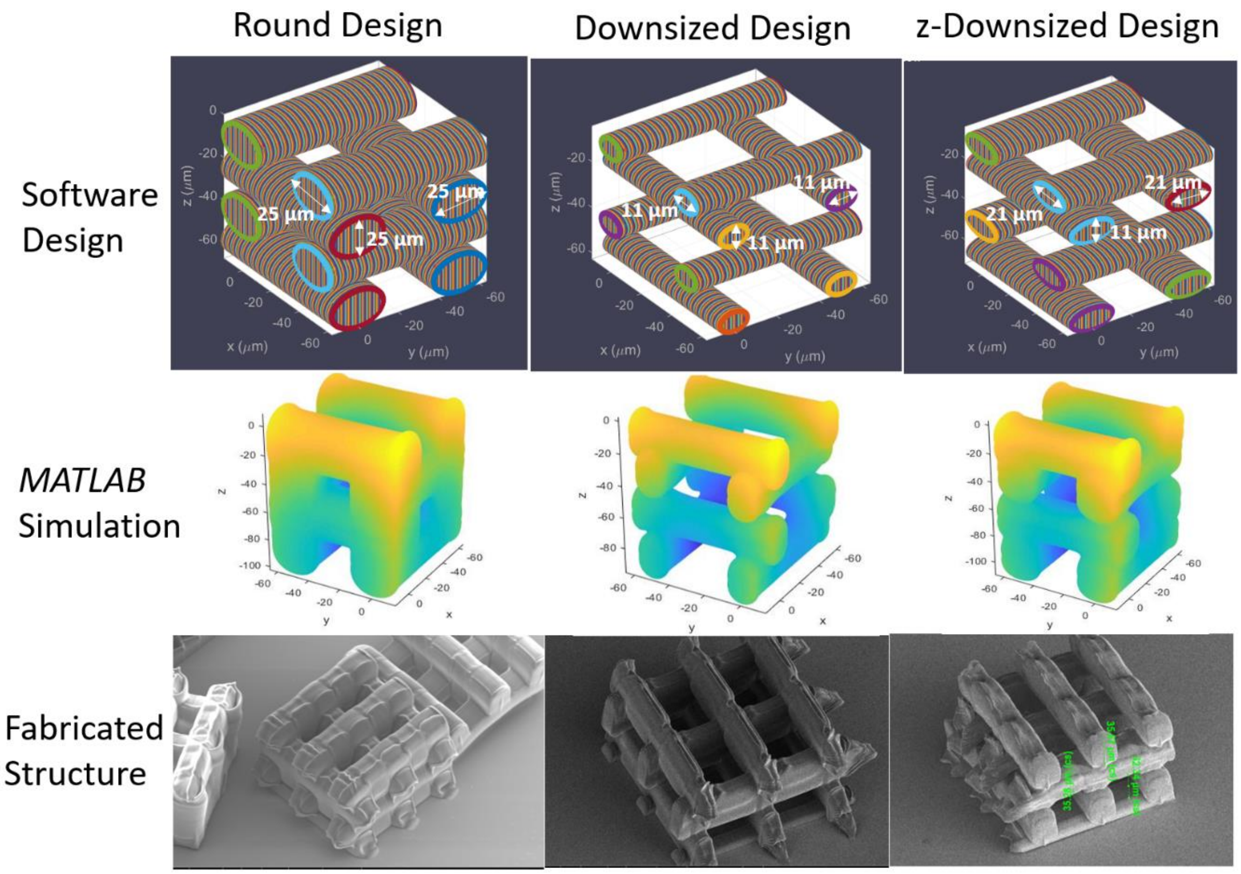

Figure S4. Effect of the same laser power in different designs: round design - 25 µm diameter beams, downsized design - 11 µm diameter beams, and z-downsized design - oval beam with 11 µm in the z - direction and 21 µm in the remaining directions. First row - design in the software program; Second row – MATLAB simulations with distance from the bottom center structure colormap to facilitate visualization; Third row – representative SEM images of PEGDA 700 fabricated structures (20 mW and 200 µm.s^{-1}). The images were taken at 45°, 10 kV, and a scale bar of 50 µm.

Cell – 3D Microstructures Interactions and Biocompatibility.

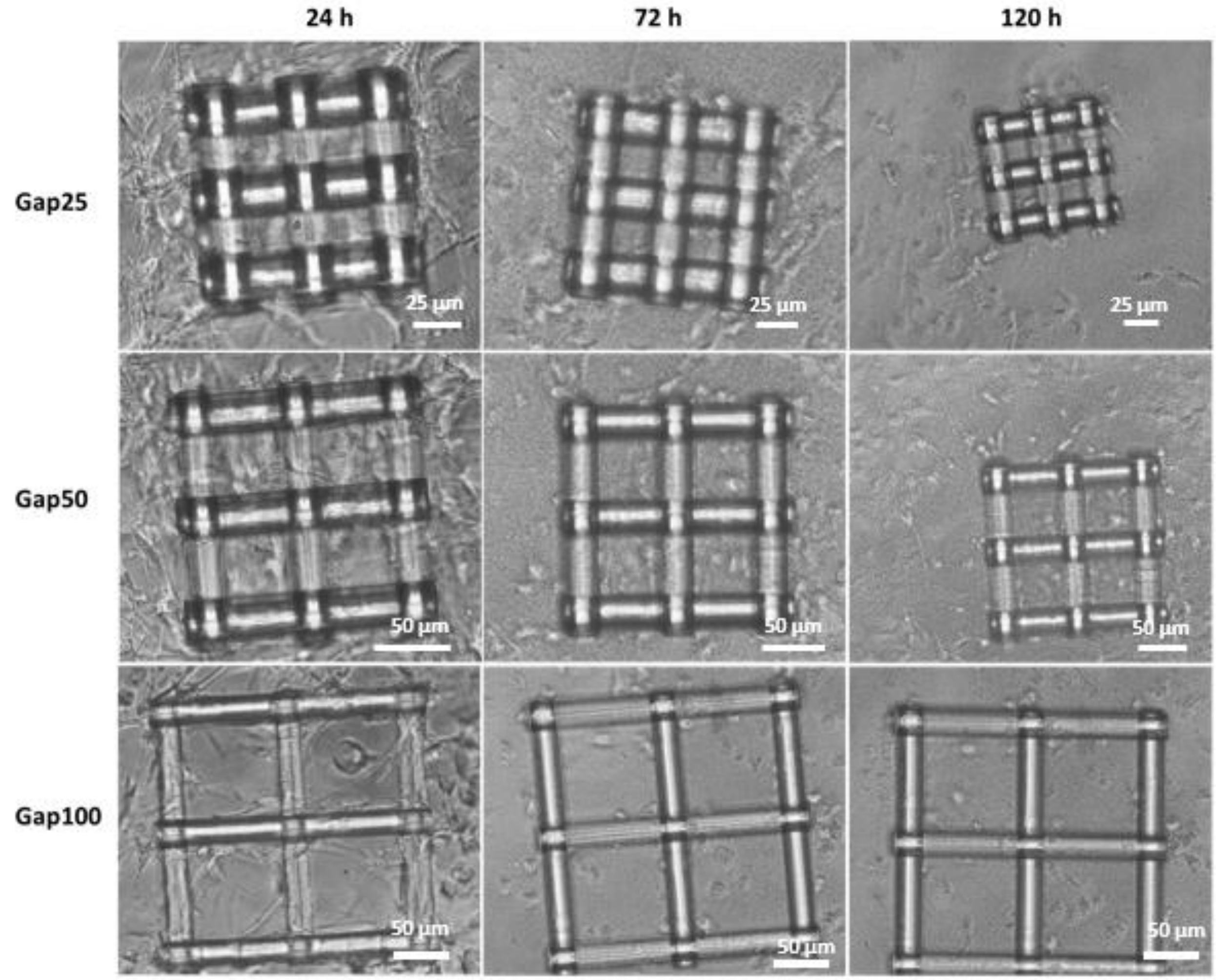

Figure S5. Top view optical microscope images of BM-MSCs interacting on IP-S woodpile scaffolds (gap 25, 50 and 100 µm) at 24, 72, and 120 h.

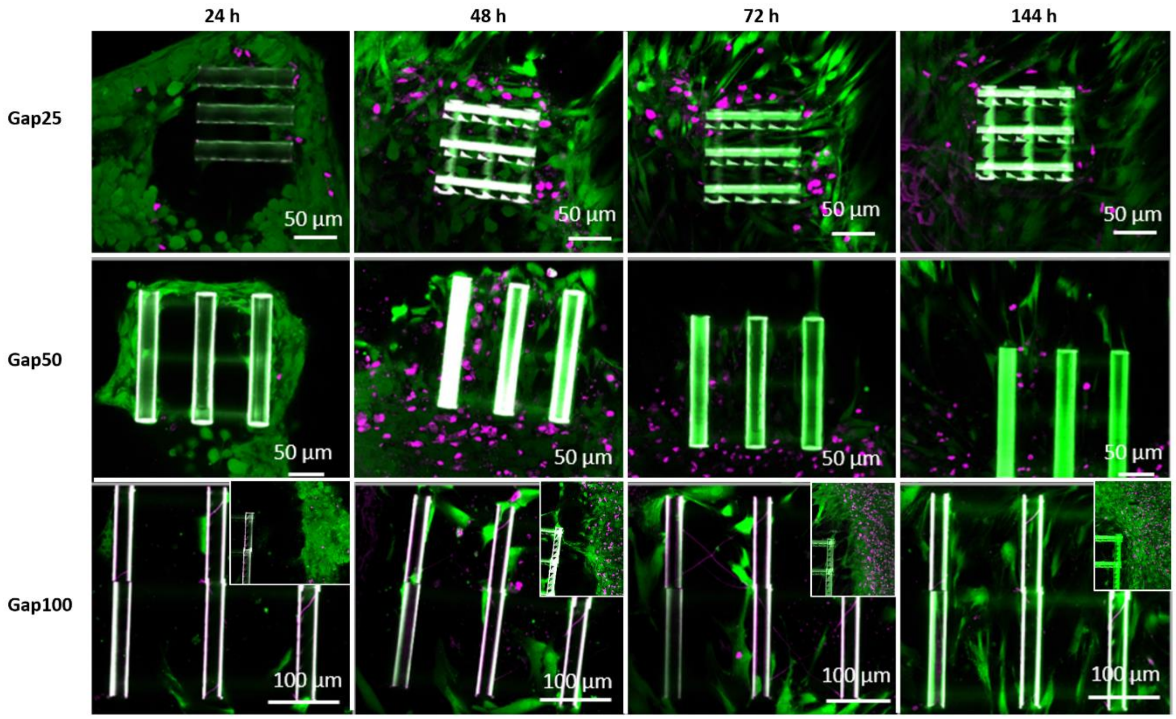

Figure S6. Effect of FBS functionalization in cell organization around the scaffolds. Confocal images of BM-MSCs cells interacting on SZ2080 woodpile scaffolds functionalized with FBS (gap 25, 50, and 100 µm) after seeding for 24, 48, 72 and 144 h. Green color – SZ2080 material/live cells; magenta color – dead cells. The insets in scaffold with 100 µm gap represent the lateral formation of agglomeration.

In Figure S6 the effect of pore sizes is compared. The pore seems to play a major role in HeLa cells interactions inside the scaffold, where pore sizes close to the cell diameter may prevent their migration to the interior. Even with UV-treated SZ2080 scaffolds, their characteristic autofluorescence is still challenging for simultaneous cell imaging in confocal microscopy. PEGDA 700 presented adhesion problems, therefore, requires further optimization to be employed in cell interaction studies.

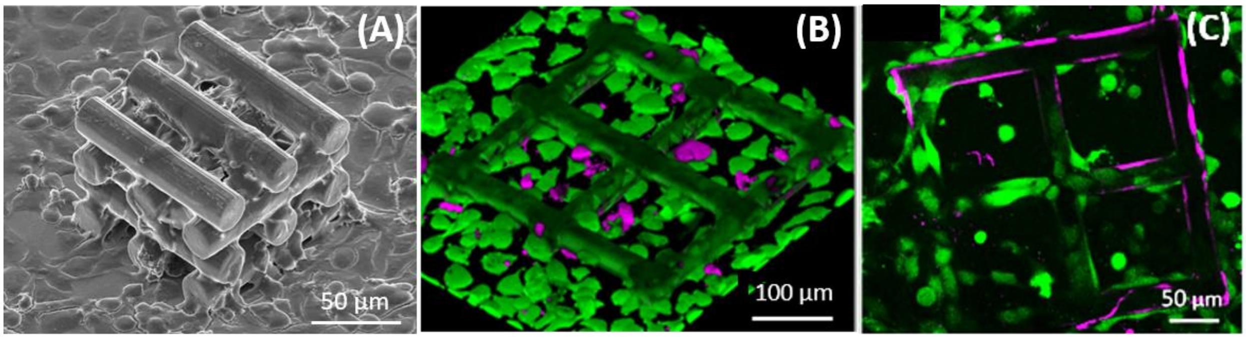

Figure S7. Representative images of HeLa cells interacting on woodpile scaffolds at 72h (A) SEM images of HeLa cells interacting on IP-S scaffolds. (B) Confocal z-stack 3D projection 45° tilted of HeLa cells interacting on SZ2080 structures functionalized with FBS. (C) Bottom view confocal images of HeLa cells interacting on PEGDA 700 scaffolds. Green color – live cells/SZ2080 material; magenta color – dead cells/PEGDA 700 material.

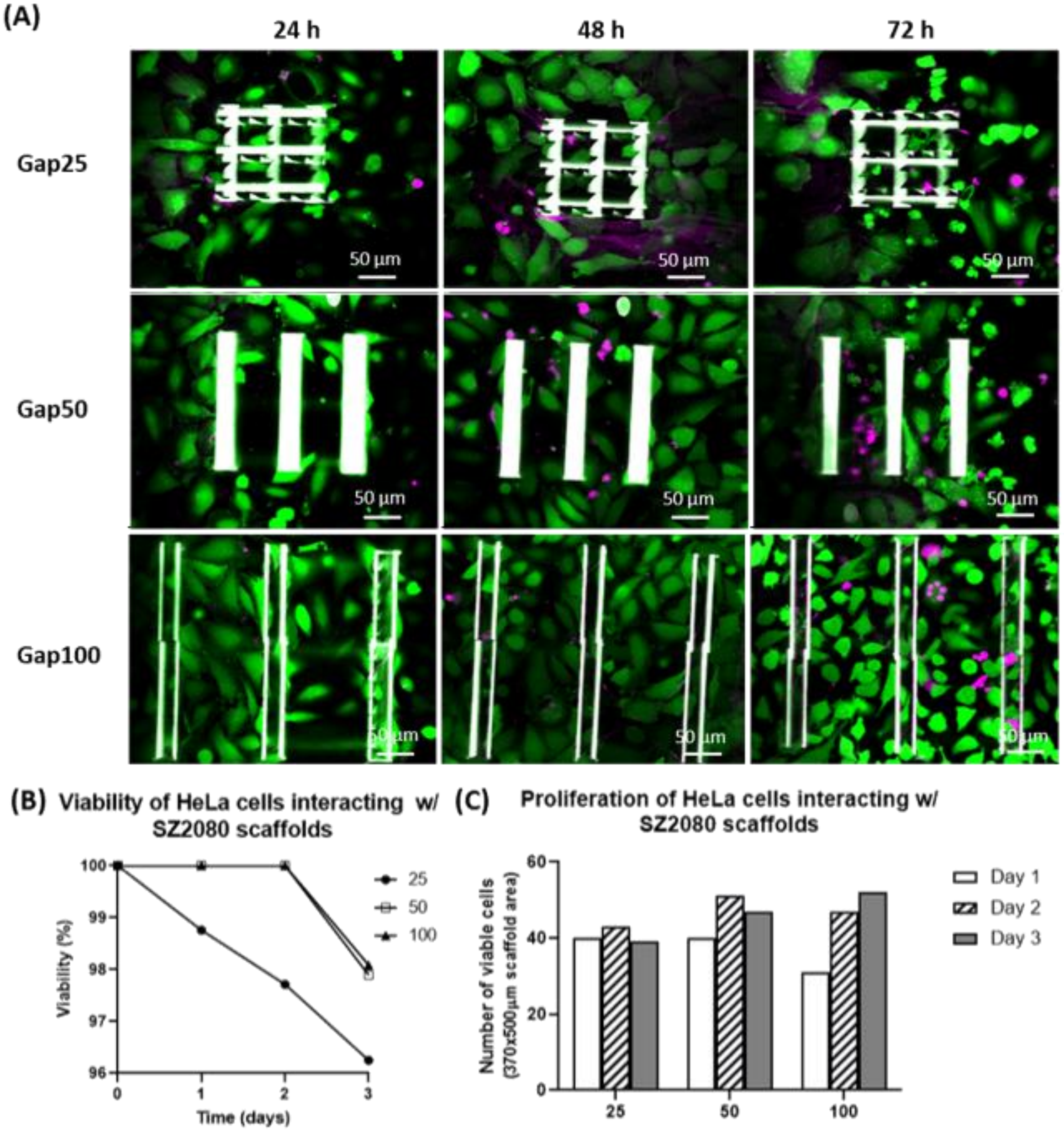

Figure S8. Hela cells interaction with SZ2080 woodpile scaffolds (gap 25, 50, and 100 μm) treated with FBS at 24, 48, and 72 h: (A) Bottom view confocal images, (B) Viability analysis, (C) Proliferation analysis. Scaffolds functionalized with FBS. Green color - live cells; magenta color - dead cells; white color – SZ2080 material; labels - number indicates scaffold gap.

Cells' Viability and Proliferation Analysis of BM-MSCs in Interaction with 3D Microstructures.

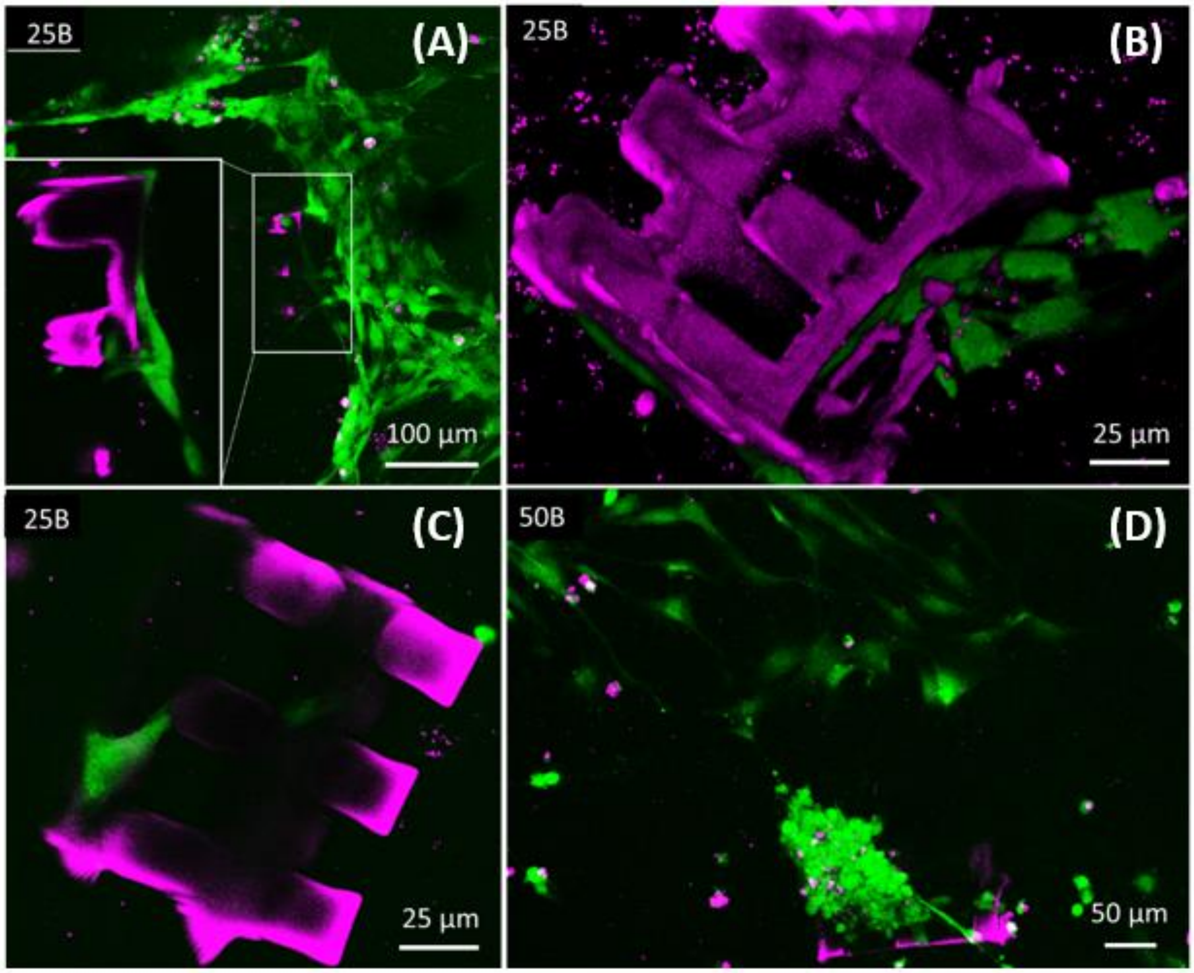

Figure S9. Top view confocal images (A and D) and 45° tilted z -stack 3D projection (B and C) of BM-MSCs cells interacting on PEGDA 700 woodpile scaffolds (gap 25B and 50B μm) at 72 h. Green color - live cells; magenta color - dead cells.
